# Dose-response to inhaled glycopyrrolate delivered with a novel Co-Suspension™ Delivery Technology metered dose inhaler (MDI) in patients with moderate-to-severe COPD

**DOI:** 10.1186/s12931-016-0426-4

**Published:** 2016-09-02

**Authors:** Leonardo M. Fabbri, Edward M. Kerwin, Selwyn Spangenthal, Gary T. Ferguson, Roberto Rodriguez-Roisin, James Pearle, Sanjay Sethi, Chad Orevillo, Patrick Darken, Earl St. Rose, Tracy Fischer, Michael Golden, Sarvajna Dwivedi, Colin Reisner

**Affiliations:** 1 Department of Medicine, University of Modena and Reggio Emilia, NOCSAE, AUSL di Modena, Via Giardini 1355, 41126 Modena, MO Italy; 2 Clinical Research Institute of Southern Oregon, Medford, OR USA; 3 American Health Research, Charlotte, NC USA; 4 Pulmonary Research Institute of Southeast Michigan, Farmington Hills, MI USA; 5 Servei de Pneumologia, Institut Clinic Respiratori, Hospital Clínic, Barcelona, Spain; University of Barcelona, Barcelona, Spain; 6 California Research Medical Group, Fullerton, CA USA; 7 University at Buffalo, State University of New York, Buffalo, NY USA; 8 Pearl Therapeutics Inc., Morristown, NJ USA; 9 Pearl Therapeutics Inc., Durham, NC USA; 10 Pearl Therapeutics Inc., Redwood City, CA USA

**Keywords:** Bronchodilators, COPD maintenance, Co-Suspension™ Delivery Technology, LAMA, Metered dose inhaler

## Abstract

**Background:**

This study forms part of the first complete characterization of the dose–response curve for glycopyrrolate (GP) delivered using Co-Suspension™ Delivery Technology via a metered dose inhaler (MDI). We examined the lower GP MDI dose range to determine an optimal dose for patients with moderate-to-severe chronic obstructive pulmonary disease (COPD).

**Methods:**

This randomized, double-blind, chronic-dosing, balanced incomplete-block, placebo-controlled, crossover study compared six doses of GP MDI (18, 9, 4.6, 2.4, 1.2, and 0.6 μg, twice daily [BID]) with placebo MDI BID and open-label tiotropium dry powder inhaler (18 μg, once daily [QD]) in patients with moderate-to-severe COPD. Patients were randomized into 1 of 120 treatment sequences. Each sequence included 4 of 8 treatments administered for 14-day periods separated by 7- to 21-day washout periods.

The primary efficacy endpoint was change from baseline in forced expiratory volume in 1 s area under the curve from 0 to 12 h (FEV_1_ AUC_0–12_) on Day 14. Secondary efficacy endpoints included peak change from baseline (post-dose) in FEV_1_ and inspiratory capacity (IC) on Days 1, 7, and 14; change from baseline in morning pre-dose trough FEV_1_ on Days 7 and 14; change from baseline in 12-h post-dose trough FEV_1_ on Day 14; time to onset of action (≥10 % improvement in mean FEV_1_) and the proportion of patients achieving ≥12 % improvement in FEV_1_ on Day 1; and pre-dose trough IC on Days 7 and 14. Safety and tolerability were also assessed.

**Results:**

GP MDI 18, 9, 4.6, and 2.4 μg demonstrated statistically significant and clinically relevant increases in FEV_1_ AUC_0–12_ compared with placebo MDI following 14 days of treatment (modified intent-to-treat population = 120). GP MDI 18 μg was non-inferior to open-label tiotropium for peak change in FEV_1_ on Day 1 and morning pre-dose trough FEV_1_ on Day 14. All doses of GP MDI were well tolerated with no unexpected safety findings.

**Conclusions:**

These efficacy and safety results support GP MDI 18 μg BID as the most appropriate dose for evaluation in Phase III trials in patients with moderate-to-severe COPD.

**Trial registration:**

ClinicalTrials.gov NCT01566773. Registered 27 March 2012.

**Electronic supplementary material:**

The online version of this article (doi:10.1186/s12931-016-0426-4) contains supplementary material, which is available to authorized users.

## Background

Symptomatic chronic obstructive pulmonary disease (COPD) is often treated in a step-wise manner, with guidelines recommending the initiation of maintenance therapy with a long-acting bronchodilator, either a long-acting β_2_-agonist (LABA) or a long-acting muscarinic antagonist (LAMA) [1]. Patients who remain symptomatic require addition of a second long-acting bronchodilator or an inhaled corticosteroid (ICS) through a combination of inhalers.

The properties associated with different inhalers may make some devices more suitable for certain patient groups than others. For example, pressurized metered dose inhalers (MDIs) are currently the most commonly used devices overall for respiratory drug delivery [2] and can be used by patients with severe airflow limitation who may struggle to activate a dry powder inhaler (DPI) [3, 4]. At the time when this study was performed, licensed LAMA and LAMA/LABA fixed-dose combination (FDC) therapies in COPD were only available via DPIs or a Soft Mist™ Inhaler (Respimat®). There is therefore an opportunity to widen the choice of inhalers available to patients by developing MDIs that can deliver a LAMA as monotherapy as well as in combination with other agents, such as a LABA and/or an ICS.

The requirement for chlorofluorocarbon-free propellant formulated MDIs has proved challenging for the creation of stable formulations and has led to the development of innovative technological advances to overcome these barriers. Co-Suspension™ Delivery Technology has evolved such that drug particles can be suspended in hydrofluoroalkane (HFA) propellant by the use of spray-dried porous particles of distearoyl-phosphatidylcholine. These particles form strong non-specific associations with the drug molecules, preventing the drugs from interacting with each other in the suspension and providing long-term stability. In analytical studies, these Co-Suspension delivery technology formulations have demonstrated excellent stability and dose uniformity, even in the nanogram dose range, with one, two, and three active ingredients formulated in a single inhaler [5].

The clinical study reported here is one of a number of studies in a Phase II program that assessed the safety and efficacy of a single-agent LAMA (glycopyrrolate [GP]) MDI, a single-agent LABA (formoterol fumarate [FF]) MDI, and dual LAMA/LABA (GP/FF [GFF]) FDC MDI, all delivered using Co-Suspension delivery technology (NCT00871182 [6], NCT01350128 [7], NCT01085045 [8], NCT01587079 [9], and NCT01349868 [10]). The LAMA, GP, delivered using Co-Suspension delivery technology as GP MDI, has demonstrated bronchodilator effects across a dose range of 4.6–36 μg in patients with COPD (NCT01350128 [7]). However, to confirm the optimum dose of GP MDI, there is a requirement to further characterize the dose–response relationship below GP MDI 4.6 μg. This study is the first assessment of the lower end of the dose–response curve for GP MDI.

This is a randomized, double-blind, chronic-dosing, placebo-controlled, multicenter crossover study to establish the dose–response curve for the GP MDI (18, 9, 4.6, 2.4, 1.2, and 0.6 μg administered twice daily [BID]) monotherapy. The aim is to provide additional support for the selection of the optimal dose of GP MDI to carry forward in studies investigating the LAMA/LABA MDI FDC in patients with COPD.

## Methods

### Patients

Male and female patients of 40–80 years of age with a diagnosis of COPD as defined by the American Thoracic Society (ATS) [11] and a smoking history of at least 10 pack-years were included in the study. Key lung function criteria for inclusion were pre- and post-short-acting bronchodilator (ipratropium bromide; Atrovent® HFA) forced expiratory volume in 1 s (FEV_1_)/forced vital capacity (FVC) ratio <0.7, post-bronchodilator FEV_1_ ≥30 % and <80 % of the predicted value and ≥750 mL at screening (Visit 1), and a pre-bronchodilator FEV_1_ <80 % at randomization (Visit 2) calculated using the Third National Health and Nutrition Examination Survey (NHANES III) reference equations [12]. Key exclusion criteria were diagnosis of asthma, α_1_-antitrypsin deficiency, or any other respiratory disease. Poorly controlled COPD that had required hospitalization or treatment with systemic corticosteroids within 3 months or antibiotics within 6 weeks prior to screening (Visit 1) also led to exclusion. In addition, patients with clinically significant abnormal electrocardiogram (ECG) results; pregnant or lactating women; and patients who could not meet ATS criteria for acceptable spirometry results were excluded [13].

Patients using oral β-agonists, inhaled LABAs, LABA/ICS combination inhalers, phosphodiesterase inhibitors, mast cell stabilizers, leukotriene antagonists, or tiotropium, discontinued these for the duration of the trial and instead received open-label ipratropium four times daily during the run-in period. Patients using ICS/LABA FDC inhalers who had received a stable dose for at least 4 weeks prior to screening were switched to the corresponding dose of a single ICS agent, such as fluticasone, mometasone or budesonide administered BID for the remainder of the study. Patients receiving a maintenance dose of an ICS that was not administered as a FDC were permitted to continue, provided they had been maintained on a stable dose for at least 4 weeks prior to screening.

Other prohibited medications included non-selective β-receptor antagonists, tricyclic antidepressants, monoamine oxidase inhibitors, anticonvulsants, and phenothiazines.

### Study design and treatment

This was a randomized, incomplete-block, crossover, placebo- (blinded) and active- (open-label) control study (NCT01566773), conducted at 10 sites in the USA from 11 April 2012 to 10 August 2012. Six doses of GP MDI (18, 9, 4.6, 2.4, 1.2, and 0.6 μg), administered BID for 14 days, were assessed. Investigators and patients were blinded to GP MDI and placebo MDI treatment using non-distinguishable MDIs. Open-label tiotropium (18 μg; Spiriva® HandiHaler®) DPI administered once daily (QD) was included as an active control.

In this study, GP is expressed as the salt, glycopyrrolate (glycopyrronium bromide), where a dose of 18 μg is equivalent to 14.4 μg glycopyrronium (active moiety).

**Fig. 1:**
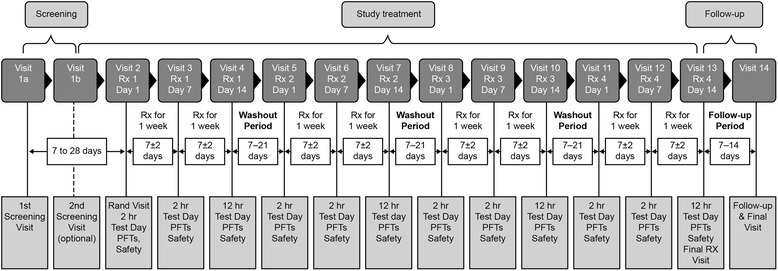
Study design schematic. *Rand* Randomization; *PFT* Pulmonary Function Test; *Rx* Treatment

Following screening, patients were randomized using an interactive web response system to one of 120 pre-defined treatment sequences, comprising four out of the eight possible treatments. Each treatment period was 14 days, separated by a 7- to 21-day washout period (Fig. 1).

At each study visit and prior to performing any study procedures, patients had to confirm that they had withheld all COPD medication for at least 6 h, or the visit was rescheduled as soon as practical and within the specified visit windows. During the study, albuterol sulfate (salbutamol HFA; Ventolin®) MDI was permitted as needed for relief of symptoms. During screening and washouts between treatment periods, ipratropium bromide (Atrovent® HFA) MDI was used as maintenance medication.

This study was conducted in accordance with International Conference on Harmonization guidelines, the Declaration of Helsinki [14], and the US Code of Federal Regulations.

### Assessments

Patients attended scheduled clinic visits at screening (Visit 1), randomization (Visit 2), then on Days 1, 7, and 14 of each treatment period.

All pulmonary function tests, including FEV_1_, FVC and inspiratory capacity (IC) as defined in ATS guidelines, were performed in accordance with ATS criteria [13]. Spirometry was performed to assess lung function pre- and post-dose at each study visit. The assessed time points on Days 1 and 7 of each treatment period were 60 and 30 min pre-dose and 15, 30, 60, and 120 min post-dose. On Day 14, post-dose time points were assessed up to 12 h post-dose.

Safety evaluations included heart rate, diastolic blood pressure, and ECGs, conducted at every clinic visit. Blood samples were taken pre- and post-dose on Days 1 and 14 of each treatment period to perform laboratory assessments including hematology and blood chemistry. Adverse events (AEs) and serious AEs were documented by investigators, with paradoxical bronchospasm and dry mouth classified as events of interest.

### Endpoints

The primary objective of this study was to assess efficacy relative to placebo MDI of GP MDI. To this end, each dose of GP MDI was compared with placebo MDI on the primary efficacy endpoint; FEV_1_ area under the curve from 0 to 12 h (AUC_0–12_) relative to baseline on Day 14 of each treatment period. FEV_1_ AUC_0–12_ values were normalized by dividing by the length of time over which they were obtained (typically 12 h). The key secondary endpoints were time to onset of action (≥10 % improvement from baseline in FEV_1_) on Day 1; peak change from baseline in FEV_1_ on Days 1, 7, and 14; change from baseline in morning pre-dose trough FEV_1_ on Days 7 and 14; change from baseline in 12-h post-dose trough FEV_1_ on Day 14; peak change from baseline in IC on Days 1, 7, and 14; mean change from baseline in morning pre-dose trough IC on Days 7 and 14; and the proportion of patients achieving ≥12 % improvement in FEV_1_ on Day 1.

### Statistical analysis

The planned sample size was 120 patients, designed to provide approximately 93 % power to detect differences of 100 mL in FEV_1_ AUC_0–12_. The 100-mL difference was selected on the grounds that it is the minimum clinically important difference, defined as the change in FEV_1_ that can be perceived by the patient [15]. The principal population for primary efficacy analyses was the modified intent-to-treat (mITT) population, comprising all patients who completed at least two treatment periods with at least 2 h of post-dose data for Day 14 from both periods. For the primary efficacy analysis of assessing the dose–response curve, the family-wise Type I error was not controlled for multiplicity beyond specifying a primary endpoint and the six key comparisons, namely each dose of GP MDI compared with placebo MDI. To compare each dose of GP MDI with placebo MDI, a linear mixed-effects model was used with FEV_1_ AUC_0–12_ as the dependent variable, and baseline trough FEV_1_, bronchodilator reversibility, period, sequence, and treatment as covariates. Baseline was defined as the mean of pre-dose values obtained from the first day of each treatment cycle averaged across periods.

Secondary efficacy analysis for the primary efficacy endpoint assessed the non-inferiority of each treatment group to open-label tiotropium using a margin for clinical relevance of 100 mL. Other secondary efficacy analyses involved superiority comparisons of secondary endpoints for each treatment group versus placebo MDI and non-inferiority comparisons versus open-label tiotropium. Non-inferiority was only determined for a treatment group if the lower bound of the 95 % confidence interval (CI) for the difference was above −100 mL and if all higher dose levels were statistically significantly non-inferior to open-label tiotropium. Non-inferiority testing was not performed for time to onset of action on Day 1, for which cumulative incidence Kaplan–Meier curves were plotted. The proportion of patients achieving ≥12 % improvement from baseline on Day 1 was tabulated and a logistic regression was used to compare treatments.

For safety analyses the safety population was used, defined as all patients who were randomized and received at least one dose of study treatment and had a post-baseline safety assessment for that treatment. Safety and tolerability data, including laboratory parameters, vital signs and ECG results were summarized descriptively, with AEs tabulated according to severity, relationship to study drug and the Medical Dictionary for Regulatory Activities system level and preferred term.

## Results

### Patient disposition and baseline characteristics

**Fig. 2:**
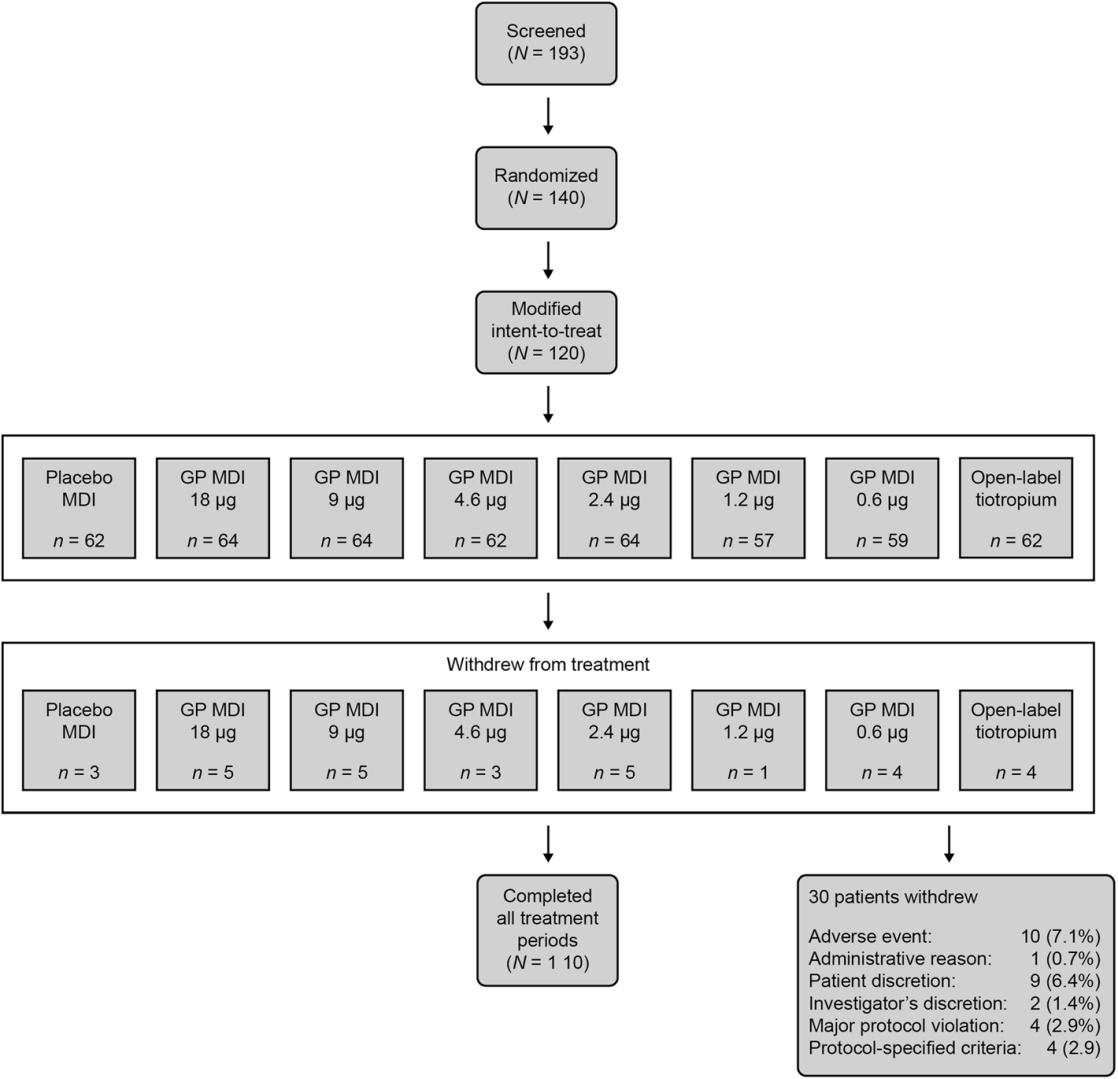
Patient disposition figure. Patients were randomized into 1 of 120 treatment sequences. Each sequence included four out of the eight possible treatments administered for 14-day periods separated by 7- to 21-day washout periods. *GP* glycopyrrolate; *MDI* metered dose inhaler

Overall, 140 patients were randomly assigned to treatment groups, and 110 (79 %) patients completed four treatment periods with 120 included in the mITT population (Fig. 2). A total of 30 patients withdrew from the study, with AEs the most common reason cited for discontinuation (Fig. 2). There were no clinically relevant differences in smoking status, disease duration, or airway limitation between the treatment groups. Overall, the majority of patients were current smokers (61 %) with a mean COPD duration of 7 years (Table 1). Airway reversibility at baseline ranged from 0.272 L (open-label tiotropium) to 0.206 L (GP MDI 9 μg [Table 1]).

**Table 1:** Patient demographics and characteristics (ITT/safety population)

Parameter	GP MDI						Placebo *n* = 62	Open-label tiotropium *n* = 62
	18 μg *n* = 64	9 μg *n* = 64	4.6 μg *n* = 62	2.4 μg *n* = 64	1.2 μg *n* = 57	0.6 μg *n* = 59		
Age, years								
Mean (SD)	61.6 (8.1)	60.6 (8.6)	60.8 (8.5)	60.2 (9.0)	61.6 (7.9)	60.3 (8.9)	61.3 (8.3)	60.7 (7.1)
Gender, *n* (%)								
Male	30 (46.9)	33 (51.6)	29 (46.8)	34 (53.1)	34 (59.6)	33 (55.9)	33 (53.2)	33 (53.2)
Race, *n* (%)								
White	58 (90.6)	58 (90.6)	57 (91.9)	58 (90.6)	53 (93.0)	55 (93.2)	56 (90.3)	57 (91.9)
Smoking status, *n* (%)								
Current	37 (57.8)	35 (54.7)	42 (67.7)	37 (57.8)	34 (59.6)	40 (67.8)	40 (64.5)	40 (64.5)
Duration of COPD, years								
Mean (SD)	7.2 (7.3)	6.0 (6.1)	8.0 (7.7)	6.6 (6.0)	6.7 (7.5)	7.5 (5.9)	8.3 (7.7)	7.2 (5.3)
Total CAT score								
Mean (SD)	20.0 (6.7)	20.7 (6.4)	19.5 (6.0)	19.8 (6.7)	20.5 (7.3)	19.9 (6.3)	20.3 (6.4)	20.5 (6.5)
*n* ^a^	59	59	61	60	55	56	60	57
Mean FEV_1_, % predicted (SD)^a^								
Pre-bronchodilator	47.0 (11.9)	46.6 (13.0)	47.0 (11.3)	46.4 (11.7)	46.2 (10.6)	45.8 (11.9)	45.6 (11.6)	43.4 (10.9)
Post-bronchodilator	55.2 (12.6)	53.7 (12.7)	54.2 (11.7)	55.1 (11.5)	54.5 (12.1)	52.9 (11.9)	53.1 (12.7)	52.5 (11.7)
Change from pre- to post-bronchodilator	19.2 (16.9)	17.9 (17.2)	16.8 (14.4)	21.0 (15.8)	18.9 (15.3)	17.5 (16.4)	18.0 (16.6)	22.5 (15.2)^b^
Mean FEV_1_, L (SD)^a^								
Pre-bronchodilator	1.363 (0.392)	1.395 (0.490)	1.409 (0.432)	1.408 (0.466)	1.369 (0.366)	1.384 (0.456)	1.358 (0.417)	1.313 (0.416)
Post-bronchodilator	1.603 (0.432)	1.601 (0.472)	1.632 (0.493)	1.670 (0.484)	1.609 (0.400)	1.602 (0.478)	1.587 (0.486)	1.585 (0.450)
Change from pre- to post-bronchodilator	0.239 (0.199)	0.206 (0.203)	0.224 (0.183)	0.261 (0.170)	0.240 (0.181)	0.218 (0.225)	0.229 (0.216)	0.272 (0.162)^b^

^a^mITT population; ^b^ *n* = 55

*CAT* COPD Assessment Test; *COPD* chronic obstructive pulmonary disease; *FEV*_*1*_ forced expiratory volume in 1 s; *GP* glycopyrrolate; *ITT* intent-to-treat; *MDI* metered dose inhaler; *mITT* modified intent-to-treat; *SD* standard deviation

### Efficacy analyses

All GP MDI doses (except 0.6 μg) showed a similar-shaped profile for FEV_1_ improvement over time on Day 14, with an early onset of action and peak treatment effect within 1–2 h post-dose (See Additional file 1: Figure S1).

**Fig. 3:**
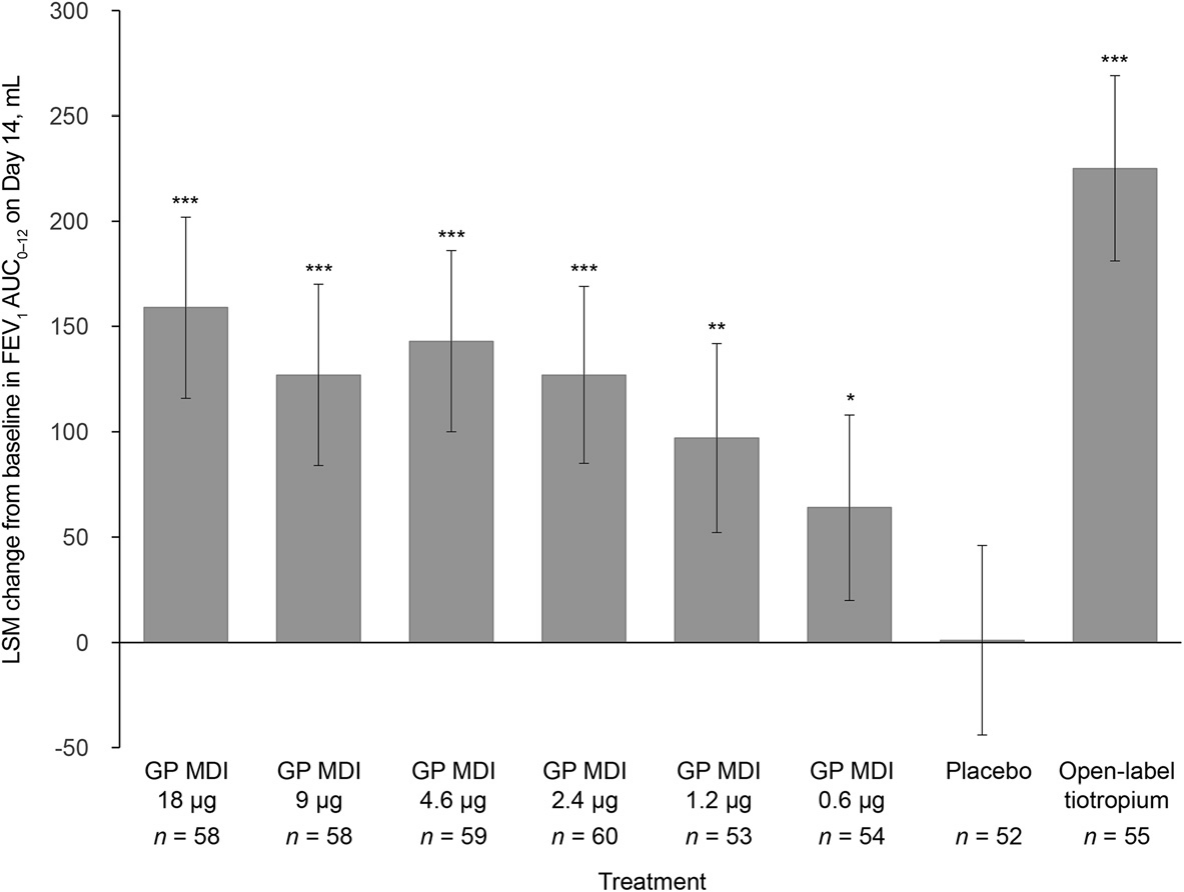
Primary endpoint: LSM change from baseline in FEV_1_ AUC_0–12_ on Day 14 (mITT population). Primary efficacy comparison for superiority to placebo: *p < 0.05; **p < 0.001; ***p < 0.0001. *FEV*_*1*_ *AUC*_*0–12*_ forced expiratory volume in 1 s, area under the curve from 0 to 12 h; *GP* glycopyrrolate; *LSM* least squares mean; *MDI* metered dose inhaler; *mITT* modified intent-to-treat

Least squares mean (LSM) change from baseline in FEV_1_ AUC_0–12_ on Day 14 ranged from 64 mL with GP MDI 0.6 μg to 159 mL with GP MDI 18 μg (Fig. 3, Table 2). All doses of GP MDI demonstrated statistically significant increases in FEV_1_ AUC_0–12_ on Day 14 compared with placebo MDI (all *p* < 0.05), with clinically relevant differences versus placebo MDI for GP 18, 9, 4.6, and 2.4 μg (LSM difference 158, 126, 141, and 126 mL, respectively; *p* < 0.0001; see Additional file 1: Figure S2). Open-label tiotropium demonstrated a statistically and clinically significant increase versus placebo MDI in FEV_1_ AUC_0–12_ on Day 14 (LSM difference, 224 mL; *p* < 0.0001). No dose of GP MDI demonstrated non-inferiority to open-label tiotropium with the lower bound of the 95 % CI exceeding the clinically relevant difference of −100 mL in FEV_1_ AUC_0–12_ on Day 14 (see Additional file 1: Figure S2).

**Table 2:** Spirometry endpoints: Day 1 and Day 14 (mITT population)

	GP MDI						Placebo	Open-label tiotropium
	18 μg	9 μg	4.6 μg	2.4 μg	1.2 μg	0.6 μg		
Primary endpoint: change from baseline in FEV_1_ AUC_0–12_, mL: Day 14								
*n*	58	58	59	60	53	54	52	55
LSM	159^†^	127^†^	143^†^	127^†^	97^***^	64^*^	1	225^†^
95 % CI	116, 202	84, 170	100, 185	85, 169	52, 141	20, 108	−43, 46	182, 269
Change from baseline in morning pre-dose trough FEV_1_, mL: Day 14								
*n*	58	59	60	59	55	55	56	57
LSM	89^***^	80^***^	67^**^	77^**^	68^**^	29	−8	126^†^
95 % CI	47, 132	38, 122	26, 109	35, 118	25, 111	−14, 72	−50, 35	84, 168
Change from baseline in evening 12-h post-dose trough FEV_1_, mL: Day 14								
*n*	58	58	59	60	53	55	54	56
LSM	116^†^	69^*^	99^***^	112^†^	66^*^	55	1	164^†^
95 % CI	69, 163	22, 117	52, 145	65, 158	17, 115	7, 104	−48, 49	116, 211
Change from baseline in morning pre-dose trough inspiratory capacity, mL: Day 14								
*n*	58	59	60	60	55	54	56	56
LSM	94^*^	45	64	104^*^	69	34	9	138^**^
95 % CI	32, 156	−16, 106	3, 124	43, 165	6, 132	−30, 97	−54, 71	76, 201
Peak change from baseline in FEV_1_, mL: Day 1								
*n*	59	59	60	59	55	55	60	57
LSM	231^†^	226^†^	165^†^	170^†^	136^**^	107	57	270^†^
95 % CI	191, 272	186, 267	125, 205	130, 211	95, 177	65, 149	17, 97	229, 311
Peak change from baseline in FEV_1_, mL: Day 14								
*n*	58	59	60	60	54	54	54	57
LSM	288^†^	288^†^	287^†^	261^†^	246^***^	188	130	361^†^
95 % CI	236, 339	237, 340	236, 338	210, 312	193, 299	135, 241	77, 183	309, 413
Peak change from baseline in inspiratory capacity, mL: Day 1								
*n*	59	59	61	60	55	54	60	56
LSM	234^***^	263^†^	151	183^*^	126	82	80	343^†^
95 % CI	163, 304	193, 333	82, 219	113, 252	54, 198	9, 155	10, 150	271, 414
Peak change from baseline in inspiratory capacity, mL: Day 14								
*n*	58	59	60	60	54	54	56	56
LSM	280^†^	259^***^	288^†^	226^**^	261^***^	172	77	284^†^
95 % CI	197, 363	177, 341	206, 370	145, 308	176, 346	87, 256	−6, 161	200, 368

Adjusted difference versus placebo: *p < 0.05; **p < 0.01; ***p < 0.001; ^†^ *p* < 0.0001

*AUC0–12* area under the curve from 0 to 12 h; *CI* confidence interval; *FEV*_*1*_ forced expiratory volume in 1 s; *GP* glycopyrrolate; *LSM* least squares mean; *MDI* metered dose inhaler; *mITT* modified intent-to-treat

**Fig. 4:**
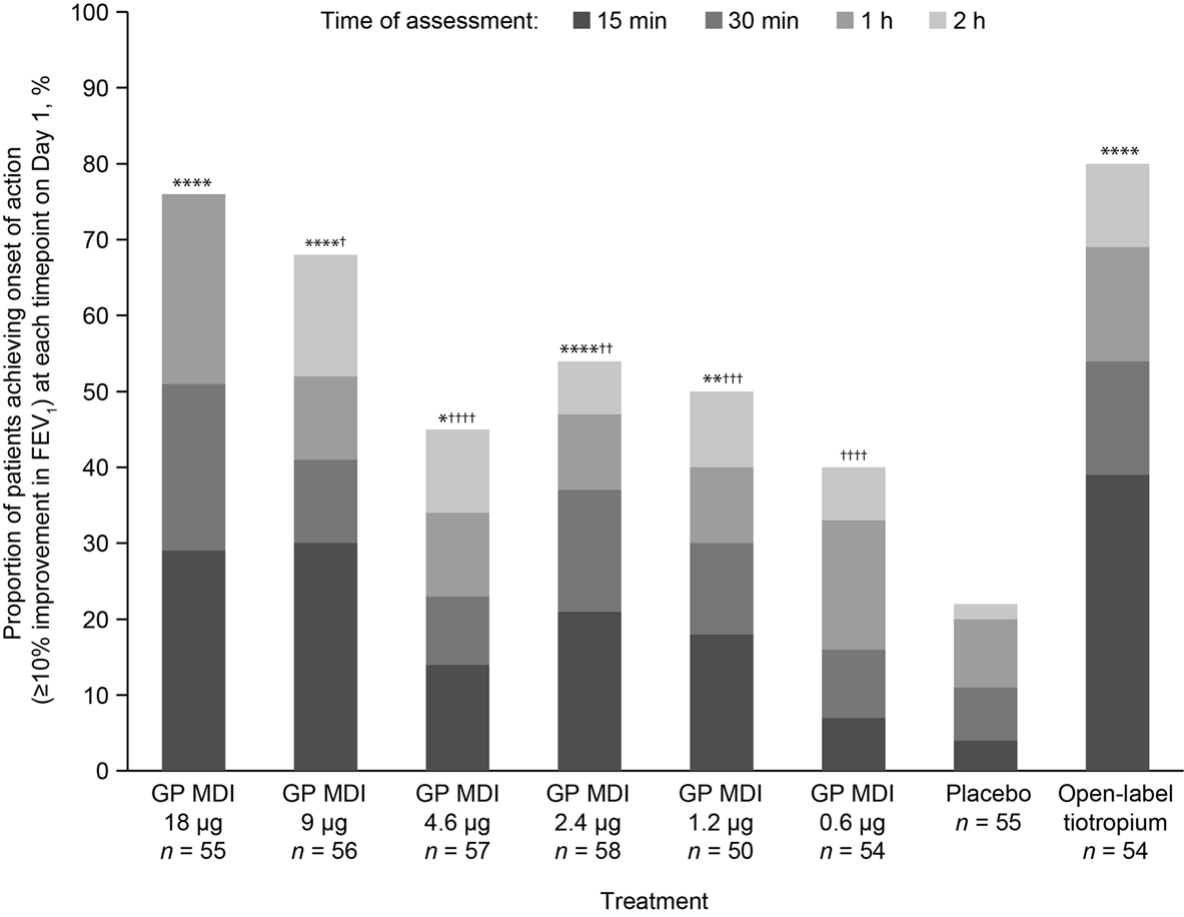
Time to onset of action (≥10 % improvement from baseline FEV_1_) on Day 1 (mITT population). Cumulative data are shown. Adjusted difference versus placebo: *p < 0.05; **p < 0.01; ***p < 0.001; ****p ≤ 0.0001. Adjusted difference versus open-label tiotropium: ^†^ *p* < 0.05; ^††^ *p* < 0.01; ^†††^ *p* < 0.001; ^††††^ *p* ≤ 0.0001. Time to onset was defined as the first post-baseline time when a ≥10 % improvement in FEV_1_ was seen relative to baseline FEV_1_, where baseline was defined as the mean of evaluable 60- and 30-min pre-dose values across Visits 2, 5, 8, and 11. *P*-values were obtained using the Murray method to account for correlation between the times to onset observed in the same subject at different periods. *FEV*_*1*_ forced expiratory volume in 1 s; *GP* glycopyrrolate; *MDI* metered dose inhaler; *mITT* modified intent-to-treat

For time to onset of action (≥10 % improvement in mean FEV_1_) on Day 1 (Fig. 4), all doses of GP MDI except 0.6 μg demonstrated a significantly faster onset of action compared with placebo MDI. The proportion of patients who had achieved onset of action by 15 min post-dose ranged from 7 % with GP MDI 0.6 μg to 30 % with GP MDI 9 μg (29 % with GP MDI 18 μg), and 39 % for open-label tiotropium, compared with 4 % for placebo MDI.

Table 2 shows the secondary endpoints on Day 1 and Day 14 across the GP MDI dose range 0.6–18 μg BID. GP MDI 18 μg was superior to placebo MDI for all secondary endpoints except for change in morning pre-dose trough FEV_1_ on Day 7 (data not shown). Treatment differences for GP MDI 18 μg versus placebo MDI were above the pre-defined threshold of 100 mL for peak change from baseline in FEV_1_ on Days 1, 7, and 14. GP MDI 9, 4.6, and 2.4 μg were often significantly superior to placebo MDI with regard to changes in lung function parameters; however, significance was less common for GP MDI 1.2 μg and, particularly, 0.6 μg versus placebo MDI.

**Fig. 5:**
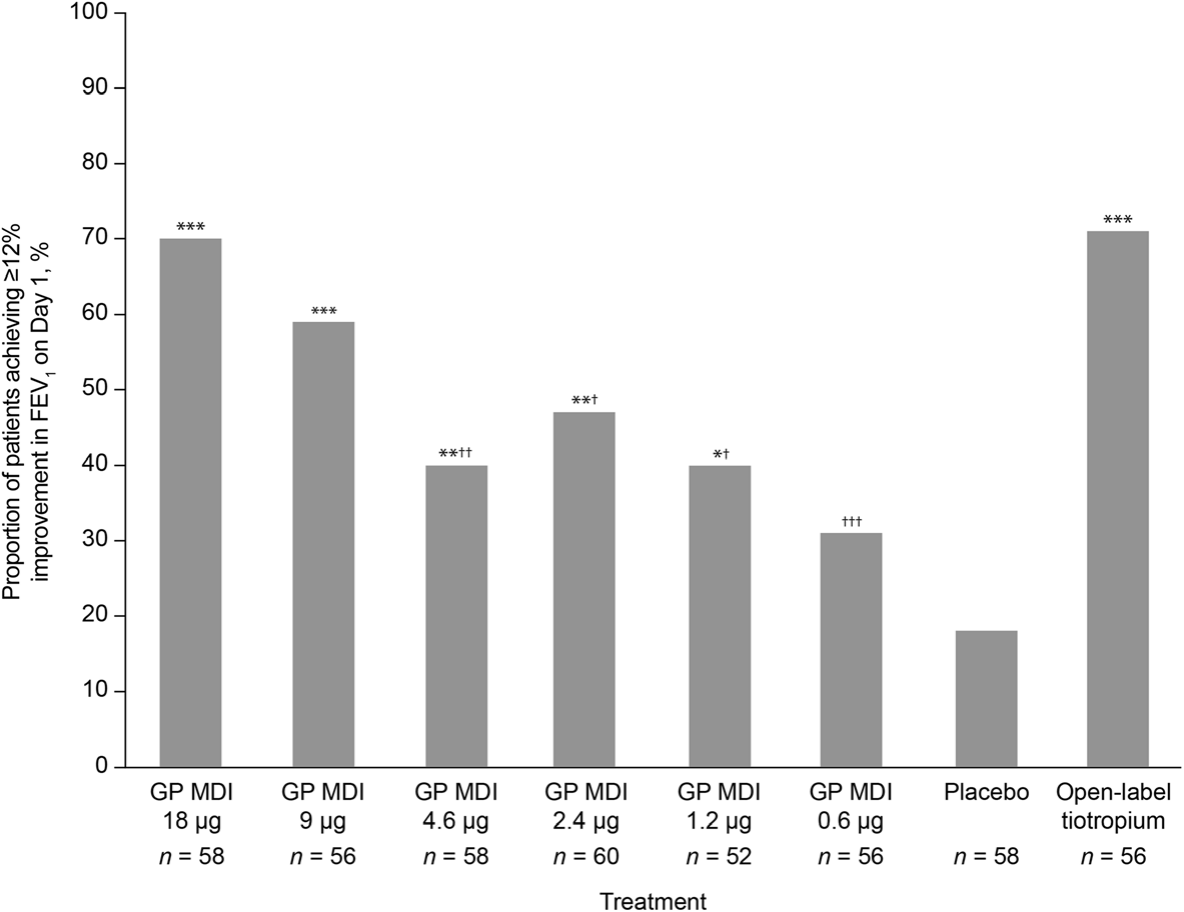
Proportion of patients achieving ≥12 % improvement in FEV_1_ on Day 1 (mITT population). Treatment difference versus placebo: *p < 0.05; **p < 0.01; ***p < 0.0001. Treatment difference versus open-label tiotropium: ^†^ *p* < 0.01; ^††^ *p* < 0.001; ^†††^ *p* < 0.0001. Estimated percentages, differences between percentages, and *p*-values were based on a logistic regression model with achievement of ≥12 % improvement in FEV_1_ as binary response and the following factors in the model: baseline FEV_1_, bronchodilator reversibility at Screening, period, and treatment. Exchangeable correlation between responses of the same subject at different periods was assumed. *FEV*_*1*_ forced expiratory volume in 1 s; *GP* glycopyrrolate; *MDI* metered dose inhaler; *mITT* modified intent-to-treat

GP MDI 18 μg consistently showed superior improvements in lung function compared with the lower doses of GP MDI, but there was no clear dose–response amongst the lower doses for many of the secondary endpoints (Table 2). GP MDI 18 and 9 μg were non-inferior to open-label tiotropium for peak change from baseline in FEV_1_ on Day 1, but this was not replicated on Days 7 and 14. GP MDI 18 and 9 μg were non-inferior to open-label tiotropium for change from baseline in morning pre-dose trough FEV_1_, using the pre-specified margin of 100 mL, on Days 7 and 14. For the proportion of patients achieving ≥12 % improvement in FEV_1_ on Day 1 (Fig. 5), a dose–response was seen, with the exception of GP MDI 4.6 μg, with a nominally higher percentage of patients achieving ≥12 % improvement with GP MDI 18 μg compared with doses below 4.6 μg, and with GP MDI 9 μg compared with GP MDI 1.2 and 0.6 μg (all *p* < 0.05).

### Safety and tolerability

A total of 54.3 % (76/140) of patients reported a treatment-emergent AE (TEAE). The incidence of TEAEs was similar across the different doses of GP MDI, ranging from 22.0 % of patients with GP MDI 0.6 μg to 29.7 % with GP MDI 2.4 μg, and compared with 24.2 % and 25.8 % in placebo MDI and open-label tiotropium groups, respectively (Table 3). Dry mouth (3.1–9.7 %), back pain, cough, and hypertension (all <5 %) were the most commonly reported AEs (Table 4), with AEs for all GP MDI doses comparable to those reported for placebo MDI and open-label tiotropium groups. A total of five patients reported serious AEs that led to study discontinuation, including one patient in each of GP MDI 9, 2.4, and 0.6 μg, placebo MDI, and open-label tiotropium treatment arms (Table 3). All events were considered unrelated to treatment. No important trends were observed in clinical laboratory results, vital signs, and ECGs.

**Table 3:** Overview of TEAEs (safety population)

	GP MDI						Placebo *n* = 62	Open-label tiotropium *n* = 62
	18 μg *n* = 64	9 μg *n* = 64	4.6 μg *n* = 62	2.4 μg *n* = 64	1.2 μg *n* = 57	0.6 μg *n* = 59		
At least one TEAE								
*n* (%)	17 (26.6)	18 (28.1)	14 (22.6)	19 (29.7)	14 (24.6)	13 (22.0)	15 (24.2)	16 (25.8)
TEAEs related to study treatment								
*n* (%)	5 (7.8)	4 (6.3)	4 (6.5)	6 (9.4)	4 (7.0)	2 (3.4)	5 (8.1)	3 (4.8)
Serious AEs								
*n* (%)	0	1 (1.6)	0	1 (1.6)	0	1 (1.7)	1 (1.6)	1 (1.6)
TEAEs leading to study withdrawal								
*n* (%)	3 (4.7)	2 (3.1)	0	1 (1.6)	1 (1.8)	1 (1.7)	1 (1.6)	1 (1.6)

*AE* adverse event, *GP* glycopyrrolate, *MDI* metered dose inhaler, *TEAE* treatment-emergent adverse event

**Table 4:** TEAEs reported for ≥3 % of patients in a treatment group (safety population)

*n* (%)	GP MDI						Placebo *n* = 62	Open-label tiotropium *n* = 62
	18 μg *n* = 64	9 μg *n* = 64	4.6 μg *n* = 62	2.4 μg *n* = 64	1.2 μg *n* = 57	0.6 μg *n* = 59		
Dry mouth	2 (3.1)	3 (4.7)	3 (4.8)	6 (9.4)	3 (5.3)	4 (6.8)	3 (4.8)	6 (9.7)
Back pain	0	2 (3.1)	0	2 (3.1)	2 (3.5)	0	1 (1.6)	0
Cough	0	2 (3.1)	0	1 (1.6)	0	1 (1.7)	3 (4.8)	0
Hypertension	1 (1.6)	1 (1.6)	1 (1.6)	2 (3.1)	1 (1.8)	1 (1.7)	1 (1.6)	1 (1.6)
Peripheral edema	0	0	2 (3.2)	0	0	1 (1.7)	2 (3.2)	0
Sinusitis	1 (1.6)	0	0	2 (3.1)	1 (1.8)	0	0	1 (1.6)
Muscle spasm	0	1 (1.6)	0	1 (1.6)	0	2 (3.4)	0	0
Pain in extremity	0	0	1 (1.6)	0	0	2 (3.4)	1 (1.6)	0
Excoriation	0	1 (1.6)	2 (3.2)	1 (1.6)	0	0	0	0
Nasopharyngitis	1 (1.6)	0	0	0	0	0	2 (3.2)	0

*GP* glycopyrrolate; *MDI* metered dose inhaler; *TEAE* treatment-emergent adverse event

## Discussion

The results of this study identified GP 18 μg as the optimal dose that demonstrated the greatest efficacy versus placebo MDI with no accompanying increase in AEs. These results enabled the selection of GP 18 μg BID as the most appropriate dose of GP, formulated using Co-Suspension delivery technology delivered via MDI, to take forward into Phase III clinical trials. As Co-Suspension delivery technology enables uniform and reliable delivery of very low doses of GP via the MDI device [5], this study is the first to have characterized the actual dose–response curve of GP using a sub-microgram dose, which previously could not be formulated either as a MDI or a DPI. GP MDI 18 μg was generally the most effective dose versus placebo MDI, with a clear dose response from 0.6 μg to 4.6 μg, followed by a relatively flat dose–response curve at the higher doses.

The primary endpoint for this study was FEV_1_ AUC_0–12_ at Day 14 relative to baseline. While this represents the full efficacy profile for GP MDI, this endpoint only represents the first half of the efficacy profile for tiotropium DPI, where the magnitude of effect in the second 12 h has been shown to be smaller [16–20]. Nonetheless, non-inferiority was defined as a lower 95 % CI bound ≥−100 mL relative to open-label tiotropium for the primary endpoint, FEV_1_ AUC_0–12_ at Day 14 relative to baseline. The value of 100 mL was selected based on ATS/European Respiratory Society task force recommendations and studies that suggest this is the minimum difference required for clinical relevance, defined as the smallest difference in FEV_1_ that is perceived by patients as important [15, 21]. For the primary endpoint, a dose–response was observed across GP MDI doses, with GP MDI 18 μg demonstrating the largest benefit (158 mL), with even the lower bound of the 95 % CI (107 mL, 208 mL) exceeding the minimum clinically important difference. Despite this robust and clinically meaningful finding, no dose of GP MDI was statistically non-inferior to open-label tiotropium for the primary endpoint on Day 14.

We observed a higher-than-expected response with tiotropium, with a response versus placebo MDI for the primary endpoint of 224 mL, compared with 107 and 199 mL observed in Phase III studies at Week 12 and Week 6, respectively [17, 20]. Whilst a peak FEV_1_ of approximately 360 mL was observed for open-label tiotropium on Days 7 and 14 of this trial, previous studies have reported lower values for this endpoint, ranging from 240 to 280 mL [22, 23]. Perhaps noteworthy with respect to the large tiotropium response was that the tiotropium group in this study also demonstrated the largest airway reversibility at baseline.

GP MDI 18 μg consistently demonstrated superiority to placebo MDI for the secondary efficacy endpoints. GP MDI showed an early onset of action, with a peak effect 1–2 h post-dose, followed by a gradual decrease in effect over 12 h, supporting the use of BID dosing. Night-time and early-morning symptoms have been reported as a common occurrence in patients with COPD [24, 25], with sleep disturbance linked to poorer outcomes including high exacerbation frequency and poor survival [26, 27]. Bronchodilator efficacy is therefore required in the second half of the 24-h dosing period, and further studies are required to investigate the benefits conferred by BID delivery of GP MDI through a second peak of bronchodilation in the evening, in particular versus tiotropium QD where the magnitude of effect in the second 12 h has been shown to be smaller [16–20].

## Conclusions

In conclusion, in this dose-ranging study evaluating single-agent GP MDI formulated using Co-Suspension delivery technology at doses of 0.6–18 μg, GP 18 μg demonstrated a robust and clinically relevant benefit compared with placebo MDI and is the most appropriate dose of GP MDI BID to take forward into Phase III clinical trials.

## Additional file

Additional file 1: Figure S1. Adjusted change from baseline in FEV_1_ over time on Day 14 (mITT population). **Figure S2.** LSM difference in FEV_1_ AUC_0–12_ on Day 14, vs placebo MDI (mITT population). (DOCX 437 kb)

## Abbreviations

AE: Adverse event
ATS: American Thoracic Society
AUC_0–12_: Area under the curve from 0 to 12 h
BID: Twice daily
CAT: COPD Assessment Test
CI: Confidence interval
COPD: Chronic obstructive pulmonary disease
DPI: Dry powder inhaler
ECG: Electrocardiogram
FDC: Fixed-dose combination
FEV_1_: Forced expiratory volume in 1 s
FF: Formoterol fumarate
FVC: Forced vital capacity
GP: Glycopyrrolate
HFC: Hydrofluoroalkane
IC: Inspiratory capacity
ICS: Inhaled corticosteroid
LABA: Long-acting β_2_-agonist
LAMA: Long-acting muscarinic antagonist
LSM: Least squares mean
MDI: Metered dose inhaler
mITT: modified intent-to-treat
QD: Once daily
SD: Standard deviation
TEAE: Treatment-emergent adverse event
