# Supplementary material for: Dose-response to inhaled glycopyrrolate delivered with a novel Co-Suspension™ Delivery Technology metered dose inhaler (MDI) in patients with moderate-to-severe COPD
Source: Respir Res. 2016 Sep 2;17(1):109. doi: 10.1186/s12931-016-0426-4 (PMC5009486; doi:10.1186/s12931-016-0426-4)
Supplement: Additional file 1: Figure S1. — Adjusted change from baseline in FEV1 over time on Day 14 (mITT population). Figure S2. LSM difference in FEV1 AUC0–12 on Day 14, vs placebo MDI (mITT population). (DOCX 437 kb) [file 12931_2016_426_MOESM1_ESM.docx]

**Additional file 1**

**Fig. S1:** Adjusted change from baseline in FEV_1_ over time on Day 14 (mITT population)


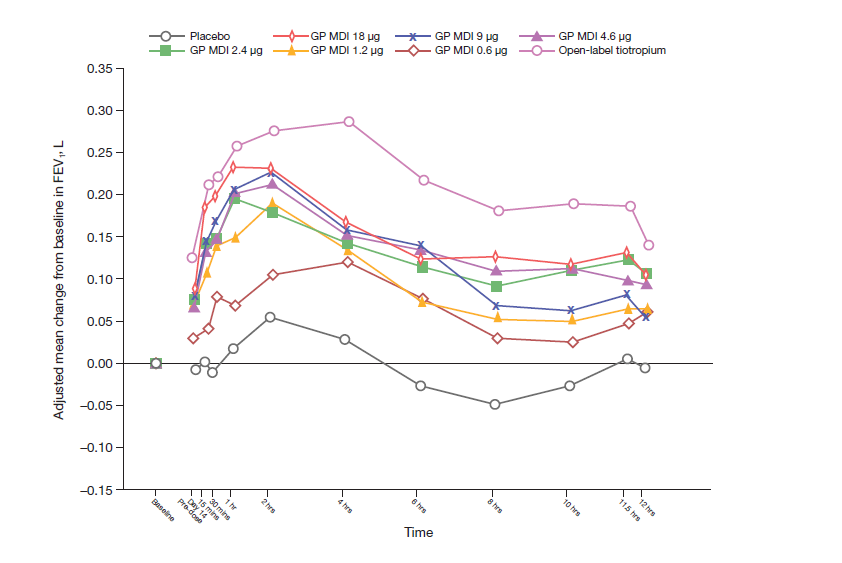


*FEV_1_* forced expiratory volume in 1 second; *GP* glycopyrrolate; *MDI* metered dose inhaler; *mITT* modified intent-to-treat

**Fig. S2:** LSM difference in FEV_1_ AUC_0–12_ on Day 14, vs placebo MDI (mITT population)


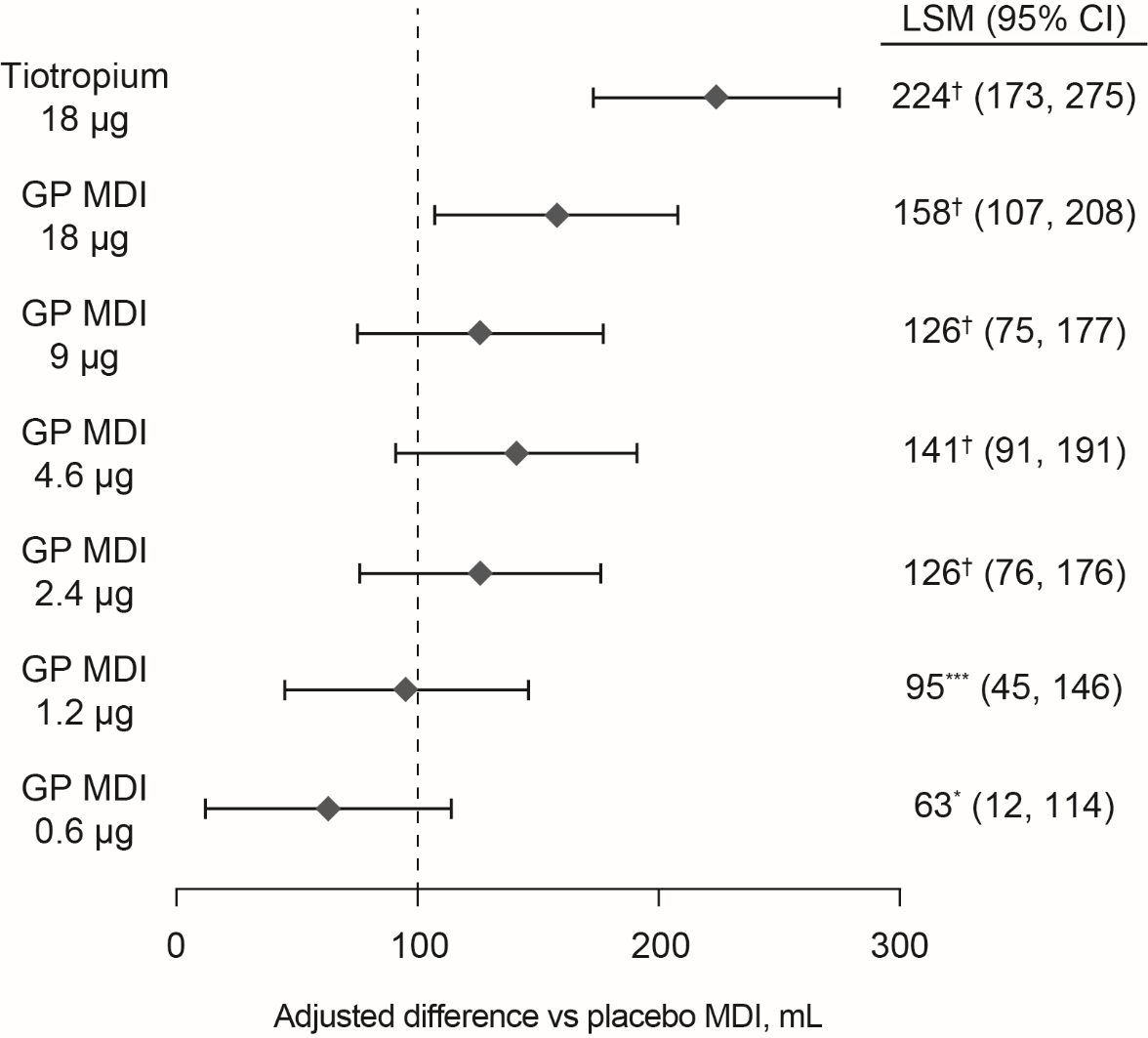


^*^ *p* < 0.05; ^**^ *p* < 0.01; ^***^ *p* < 0.001; ^†^ *p* < 0.0001

*AUC_0–12_* area under the curve from 0 to 12 hours; *CI* confidence interval; *FEV_1_* forced expiratory volume in 1 second; *GP* glycopyrrolate; *LSM* least squares mean; *MDI* metered dose inhaler; *mITT* modified intent-to-treat
